# Phytochemicals in the treatment of patients with depression: a systemic review

**DOI:** 10.3389/fpsyt.2024.1509109

**Published:** 2024-12-09

**Authors:** Natalia Picheta, Julia Piekarz, Karolina Daniłowska, Karol Mazur, Halina Piecewicz - Szczęsna, Agata Smoleń

**Affiliations:** Chair and Department of Epidemiology and Clinical Research Methodology, Medical University of Lublin, Lublin, Poland

**Keywords:** phytochemicals, depression, turmeric, lavender, saffron, St. John’s wort

## Abstract

**Background:**

Depression is a complex mental disease whose incidence increases every year; 300 million people worldwide currently suffer from it. Women are more likely to suffer from depression, twice the rate as men. It is one of the few illnesses that can lead to suicide, which makes it very dangerous – currently, 700,000 people die from suicide and it is the 4th most common cause of death in people aged 15-29. The treatment strategies for depression is a big challenge for physicians, pharmacists, scientists and classic remedies cause many side effects. Therefore, natural phytotherapy with herbs can prove to be a good solution. Phytotherapy is a popular treatment method used for centuries in Chinese medicine or Ayurveda.

**Materials and methods:**

The study conducted a comprehensive database search PubMed, ClinicalKey and MedNar covered the years 2015 - 2024 to provide the most up-to-date data. 13 randomized controlled trials and 1 meta – analysis were included in the systematic review.

**Results:**

Many plants show anti-inflammatory, antioxidant and cognitive enhancing effects, which are particularly important in depression. In the treatment of depression, plants such as *Crocus sativus L. stigma*, *Lavandula angustifolia, Hypericum perforatum L.* and *Curcuma longa L*. have proven to be effective. They show good effectiveness in human studies and alleviate the symptoms of depression. Herbal products can support classical pharmacotherapy, but this requires further research. Non-commercial clinical trials in the future should provide answers to research questions: at what stage of treatment of patients with MDD will the use of phytochemicals be most appropriate in terms of therapy efficacy and safety for the patient.

**Conclusions:**

*Crocus sativus L. stigma*, *Lavandula angustifolia, Hypericum perforatum L.* and *Curcuma longa L*. in modern medicine can help improve the well-being of patients with depression. The use of herbs as an intervention was associated with a decrease in the concentration of proinflammatory cytokines and an overall improvement in the mood of patients.

Further research should be undertaken into combining both therapies in order to improve patients’ quality of life and reduce treatment costs.

## 1 Introduction

Treating major depressive disorder (MDD) presents a challenge in modern medicine, with the number of cases are continually increasing, and current treatment methods have certain limitations (1). MDD affects 3.8% of the population, with 5% adults and it occurs 50% more frequently in women (2). Around 10% of women who have recently given birth are diagnosed with depression (3). In Europe, approximately 6.38% suffer from depression, with rates varying from 2.58% in the Czech Republic to as high as 10.3% in Iceland. In Poland, 766,000 adults have experienced at least one depressive episode in their lifetime (4). In the United States, 6.5% of young adults aged 18 and older have experienced depressive disorders, while in Latin America, depression affects 5% of the population (4). MDD is increasing year by year and currently affects 300 million people worldwide. It is predicted to become the world’s leading cause of disease by 2030 (5).

The development of depression is hypothesized to stem from disturbances in neurotransmitters, receptors, the hypothalamic-pituitary-adrenal (HPA) axis, cytokines, neuroplasticity, and systemic influences or brain-derived neurotrophic factor (BDNF) (6). These theories are developed in the context of the use of particular plants in the treatment of MDD.

Depressive disorder is described by persistent feelings of sadness, hopelessness, and worthlessness lasting for at least 2 weeks. Symptoms of depression include irritability, decreased cognitive function, loss of energy, disruptions in sleep and eating patterns, and notably, a marked reduction in interest in activities previously enjoyed by the patient. Depression poses a significant problem as its incidence is very high among the population and is inseparably linked to an increasing level of suicides (7). Because depression is a devastating disease, an important aspect at the beginning of treatment is to improve the patient’s quality of life.

The treatment of depression is based on four main methods: antidepressant medications - most commonly selective serotonin reuptake inhibitors (SSRIs) such as fluoxetine, citalopram, paroxetine or sertraline are used, or less frequently serotonin norepinephrine reuptake inhibitor (SNRI), cognitive-behavioral therapy, interpersonal psychotherapy and somatic psychotherapy, including electroconvulsive therapy in treatment-resistant depression (8).

**Figure 1:**
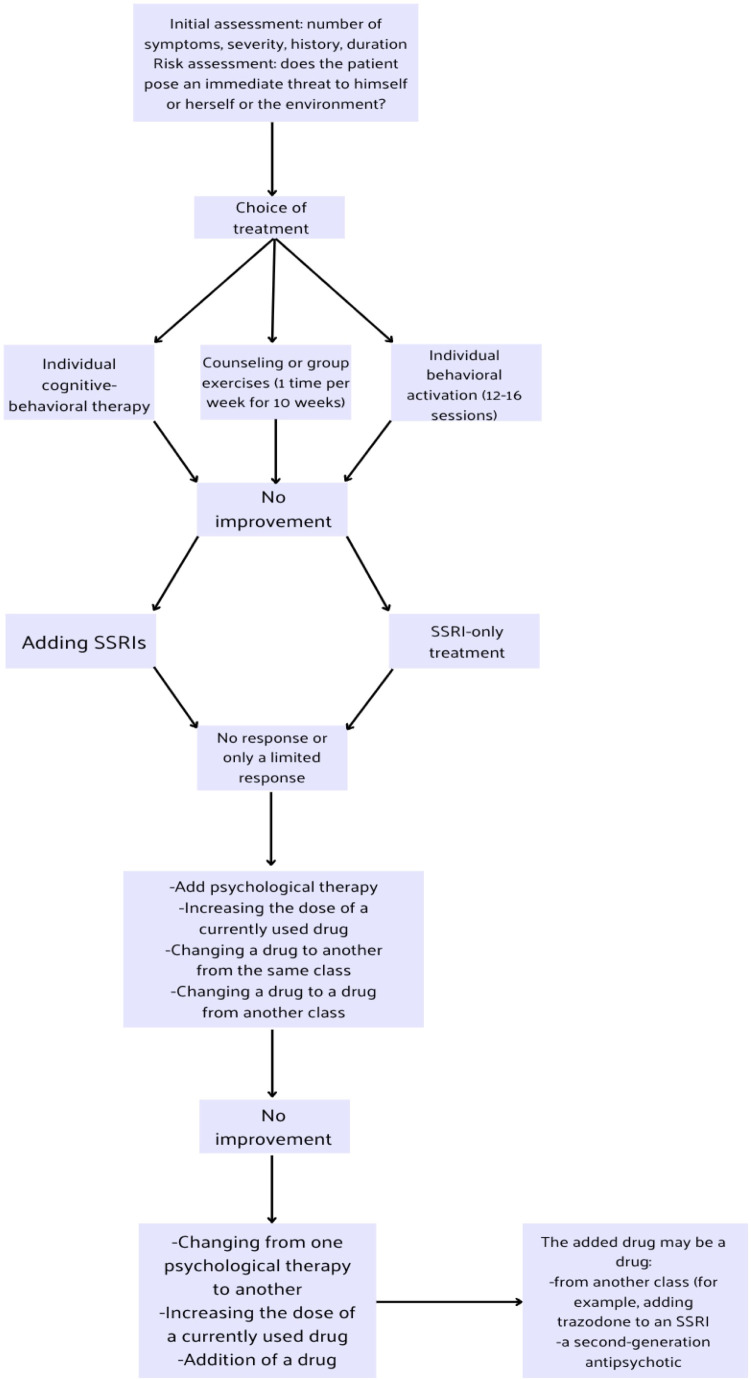
Massive depression disorders treatment algorithm based on National Institute for Health and Care Excellence (NICE) guidelines (9).

The treatment of a patient with depression should take into account the categories of depression severity and the presence of other chronic diseases in the patient. **Figure 1** illustrates the treatment scheme and its modifications during the course of unipolar depression therapy.

Classical pharmacotherapy is effective, however using in the long term can generate many side effects, such as impotence, weight gain, QT interval prolongation and photosensitivity (10). Phytochemicals with antidepressant impact may be helpful to use in depression treatment.

According to the World Health Organization (WHO), up to 170 member states use traditional medicine, and about 80% of the population chooses natural modalities for primary health care (11, 12). Approximately 40% of the medicines commonly used today are created from natural sources, including drugs used in malaria resistant to traditional treatment - artemisinin based on the active ingredient wormwood, and vincristine and vinblastine as medicines used in pediatric cancers based on the Madagascar periwinkle, or digitalis glycosides for the treatment of heart failure used since the 18th century (13, 14).

Plants such as *Crocus sativus L. stigma, Lavandula angustifolia, and Curcuma longa L.* have demonstrated therapeutic effects in the treatment of depressive disorders. Lavender (Lavandula angustifolia), through actions similar to benzodiazepines, enhances the activity of Gamma-aminobutyric acid (GABA) within the amygdala and affects the N-methyl-D-aspartate (NMDA) receptor. Saffron (*Crocus sativus L. stigma*) inhibits the reuptake of monoamines and acts antagonistically on both the GABA and NMDA receptors (15). This plant not only exerts antidepressant effects but also anxiolytic properties and has shown sedative and anticonvulsant effects as well.

Turmeric (*Curcuma longa L.)* exhibits neuroprotective effects, normalize the HPA axis, which is up-regulated in depressed patients, reduces oxidative stress, and protects mitochondria from damage. Moreover, it acts antidepressant by influencing the serotonergic system and the adenylate cyclase-cyclic adeno-sine monophosphate (AC-cAMP) pathway. Curcumin has been shown to enhance the influence of neurotransmitters such as 5-hydroxytryptamine receptors (5-HT) and to inhibit the action of monoamine oxidase A (MAO-A), in addition, it reduces inflammation and regulates the HPA axis (15). Another herb in question is St. John’s wort (*Hypericum perforatum L*.), which also inhibits MAO activity and contributes to maintaining brain neuroplasticity.

All the above-mentioned plants help to inhibit the progression of depression at all known levels of its development, and thus may contribute to improving the well-being of patients. The plan of our study was to gather and analyze researches on the use of medicinal herbs in the therapy of depression. Furthermore, overview of available researches allowed for a better understanding of the impact of medicinal plants on receptors and neurotransmitters in the central nervous system.

## 2 Methods

### 2.1 Review desing and search strategy

The research focus on comparing these therapies with conventional treatments. The PICO model was used to organize and guide the literature review.

- Population: the target population consists of patients suffering from depression.
- Intervention: Interventions include the use of herbal therapies such as turmeric, saffron, lavender, St. John’s Wort. These herbs were selected based on their traditional and contemporary use in treating symptoms of depression.
- Comparison: The effectiveness and safety of herbal therapies were compared with conventional treatments for depression. This comparison will allow an assessment of the relative benefits and risks of herbal therapies.
- Outcome: Outcomes analyzed include the effectiveness and safety of herbal therapies in the treatment of depression. Effectiveness measured by reduction of depression symptoms and overall health benefits. Additionally, potential side effects associated with the use of herbal therapies will be assessed.

The search strategy used the most important inclusion criterion: the selection of primary studies of the RCT type and secondary studies - meta-analyses or systematic reviews provided that they do not include the results of RCTs found in databases as primary studies. The focus was on these types of studies because they offer the least risk of bias and provide high credibility of scientific evidence. Case studies, animal studies and other studies were dropped. No less important criterion for the inclusion of studies was the time of publication of these studies (last 10 years).

The search terms in the databases: PubMed, ClinicalKey and MedNar covered the years 2015 - 2024 to provide the most up-to-date data. Keywords used: “phytochemicals”; “depression”; “turmeric”; “lavender”; “saffron”; “St. John’s Wort”. Manual searching of retrieved studies was performed not to miss randomized controlled trials (RCTs). Two authors (N.P. & J.P.) independently explored databases and carefully evaluated the articles. Inconsistent issues were resolved by discussion with the other two authors (K.D. & H.S.).

### 2.2 Collecting data

The review included 1 meta-analysis, 13 randomized trials with 1050 patients in which herbal preparations were administered in the intervention group. The RCTs provided sufficient data on the baseline and final wellbeing of patients with depression based on appropriate patient questionnaires.

### 2.3 Selection and identifications of studies

**Figure 2:**
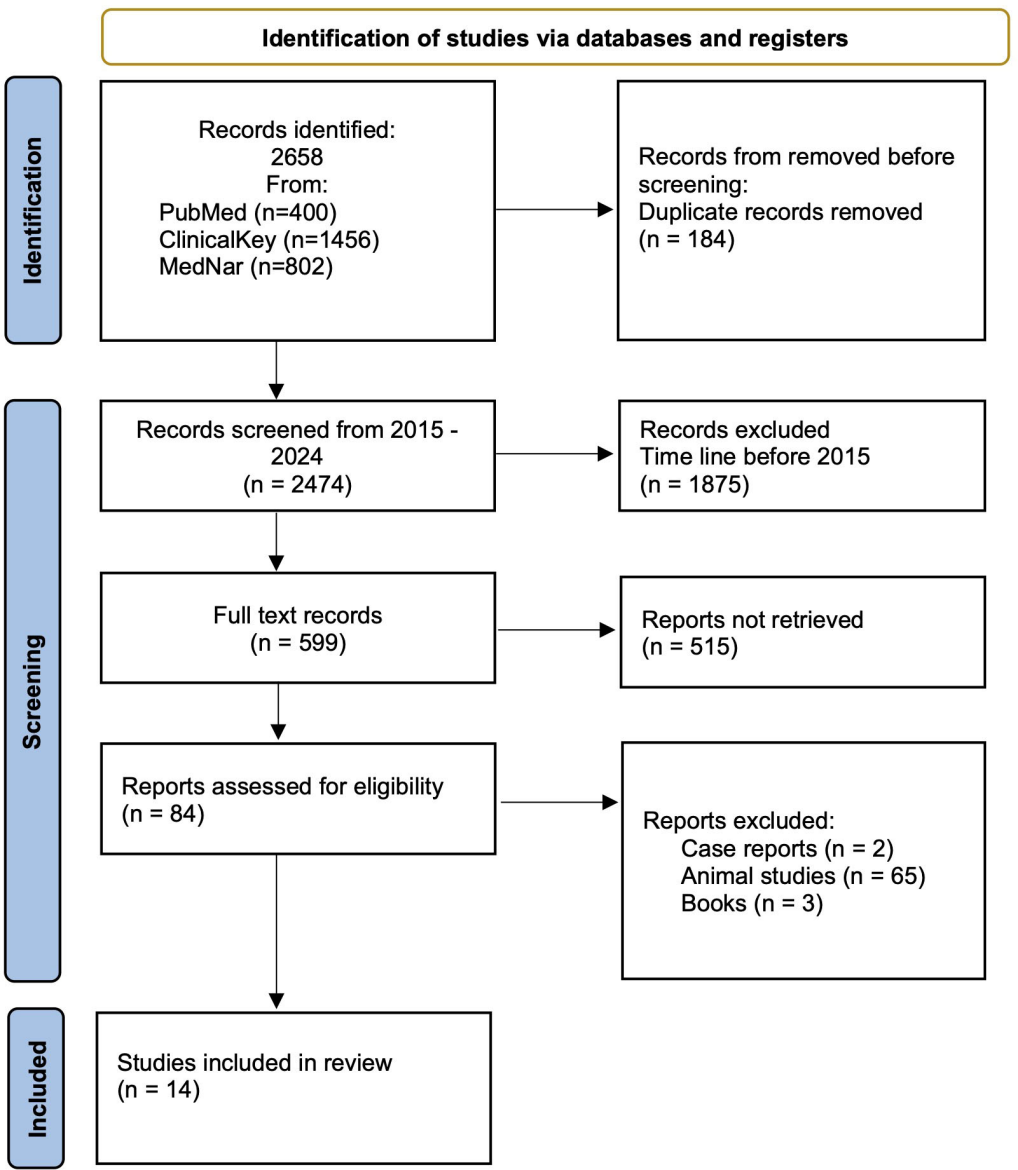
Preferred Reporting Items for Systematic Reviews and Meta-analyses (PRISMA) flow diagram of study identification, inclusion, and exclusion.

A systematic search of databases yielded 2658 records. 184 duplicate records were removed. 1875 records that did not meet the time criteria and 515 records that did not meet the criteria were excluded due to: lack of significance of the results or outcomes. Another 69 results were excluded from the study because they were animal studies, case repots or book fragments. Ultimately, 14 papers were included in the main analysis. After evaluating the quality of included studies, 14 studies were classified as good quality. The detailed data selection and identification process is presented in **Figure 2**.

### 2.4 Assessment of risk of bias in the included studies

All included RCT were assessed risk of bias using the revised Cochrane ‘Risk of bias’ tool for randomized trials (RoB 2.0) (Higgins 2019). RoB 2.0 addresses five specific domains: (1) bias arising from the randomization process; (2) bias due to deviations from intended interventions; (3) bias due to missing outcome data; (4) bias in measurement of the outcome; and (5) bias in selection of the reported result. Two review authors (H.S. & K.M.) independently applied the tool to 13 included RCT, and recorded supporting information and justifications for judgements of risk of bias for each domain (low; high; some concerns). Any discrepancies in judgements of risk of bias or justifications for judgements were resolved by discussion to reach consensus between the two review authors, with a third review author acting as an arbiter (A.S.). Meta-analysis was assessed using a Measurement Tool to Assess Systematic Reviews (AMSTAR 2) - 16-item scale designed to appraise systematic reviews of healthcare interventions and to rate the overall confidence in their results. Two authors (H.S. & A.S.) separately assessed the methodological quality of meta-analysis.

## 3 Results

### 3.1 Herbs used in depression treatment

#### 3.1.1 Saffron

Saffron, *Crocus sativus L. stigma*, is a plant rich in safranal, crocin and crocetin. It belongs to the Iridaceae family of the genus *Crocus* (16). It is native to Asia Minor and is mainly cultivated in Iran, Greece, India, Italy and Morocco. Safranal is the terpenoid responsible for the smell of saffron. It accounts for 30 - 70% of the volatile compounds of this plant (17). Saffron has been used since ancient times as a perfume, food seasoning and colouring. In addition, in medicine, it is traditionally famous as an agent used in the treatment of many systemic pathologies: respiratory, nervous, cardiovascular or gastrointestinal (16). It exhibits anti-inflammatory and antioxidant effects, particularly effective at neutralising superoxide anions and also inhibits the action of the ATP-induced NLRP3 inflammasome responsible for the inflammatory response (18, 19). In addition, it favourably affects central nervous system function and has antitussive and nephroprotective and antihypertensive effects (17). Crocin and crocetin give saffron its yellow-red colour. These are carotenoids derived from the stigma of saffron and have both anti-inflammatory and antioxidant, anti-apoptotic and neuroprotective properties. Historically, saffron has been used to treat infertility and impotence (20). Saffron extract additionally possesses sleep-inducing, sedative, antidepressant, antiepileptic and antispasmodic properties (21).

#### 3.1.2 Lavender

Lavender, a plant used in aromatherapy, is known for its sleep-inducing and sedative properties. It is known for its anti-inflammatory and cardioprotective effects (22). Lavender is mainly cultivated for the extraction of essential oils, which contain more than 100 different substances, including terpenoid and phenolic compounds. The main substances are the monoterpenoids linalyl acetate, camphor, linalool, 1,8-cyneol, β-ocimen (23). These substances are known for their antioxidant properties, which is particularly important in the pathogenesis of depression and other chronic diseases, as oxidative stress contributes to their development (24). The plant of the genus Lavandula, which belongs to the *Lamiaceae* family, includes as many as 39 species. *Lavandula angustifolia, Lavandula x intermedia, Lavandula latifolia or Lavandula stoechas* are most commonly used in the pharmaceutical, food and cosmetic industry (25).

#### 3.1.3 Curcuma longa

*Curcuma long*a is a plant from the *Zingiberaceae* family, grown especially in Southeast Asia, mainly in India, where 829,300 tonnes are produced annually (26). Due to its yellow colour, the Indian spice is also called ‘Indian saffron’ (27). The plant is used as a food seasoning and herbal medicine (28). Toxicity studies have shown that turmeric can be used even in high doses, but not exceeding 12 g (27). Curcumin supplements are the best-selling herbal agent in the world with promising preclinical effects of their intake (29). Turmeric contains more than 300 biologically active substances. These include polyphenols, sterols, diterpenes, sesquiterpenes, triterpenoids and alkaloids (30). The main active constituents responsible for many of the activities of turmeric are the curcuminoids: curcumin (60-70%), demethoxycurcumin (20-30%) and bisdemethoxycurcumin (10 –15%), which account for only 2 to 5% of the plan (30, 31). In addition, the plant contains: diphenylalkanoids, alkaloids, flavonoid glycosides, terpenes, phenolic acids. It is attributed with the following actions: antimicrobial, anti-asthmatic, anti-inflammatory, antifungal, antioxidant, antidiabetic, antiproliferative, anticancer, hepatoprotective, nephroprotective and neuroprotective (32). Also, it is stated to have potential benefits in the treatment of ovarian insufficiency, endometriosis and polycystic ovary syndrome (30).

#### 3.1.4 Hypericum perforatum L.

*Hypericum perforatum L*., known also as John’s wort is famous for its antidepressant properties; two compounds are particularly important - hypericin and hyperforin, not forgetting prophenols and flavonoids (33). Thanks to its hypericin and hyperforin content, it has wound-healing and antibacterial properties (34). It is also said to have anti-inflammatory and anti-cancer properties. It is a herb commonly used in traditional Chinese or Greek medicine for menstrual cramps, stomach ulcers and sciatica (35). Due to the presence of the above-mentioned active compounds, the probable mechanism of action of St. John’s wort is the inhibition of the reuptake of serotonin, dopamine and noradrenaline. It has not yet been approved for its properties by the FDA, so it is only a dietary supplement.

**Figure 3:**
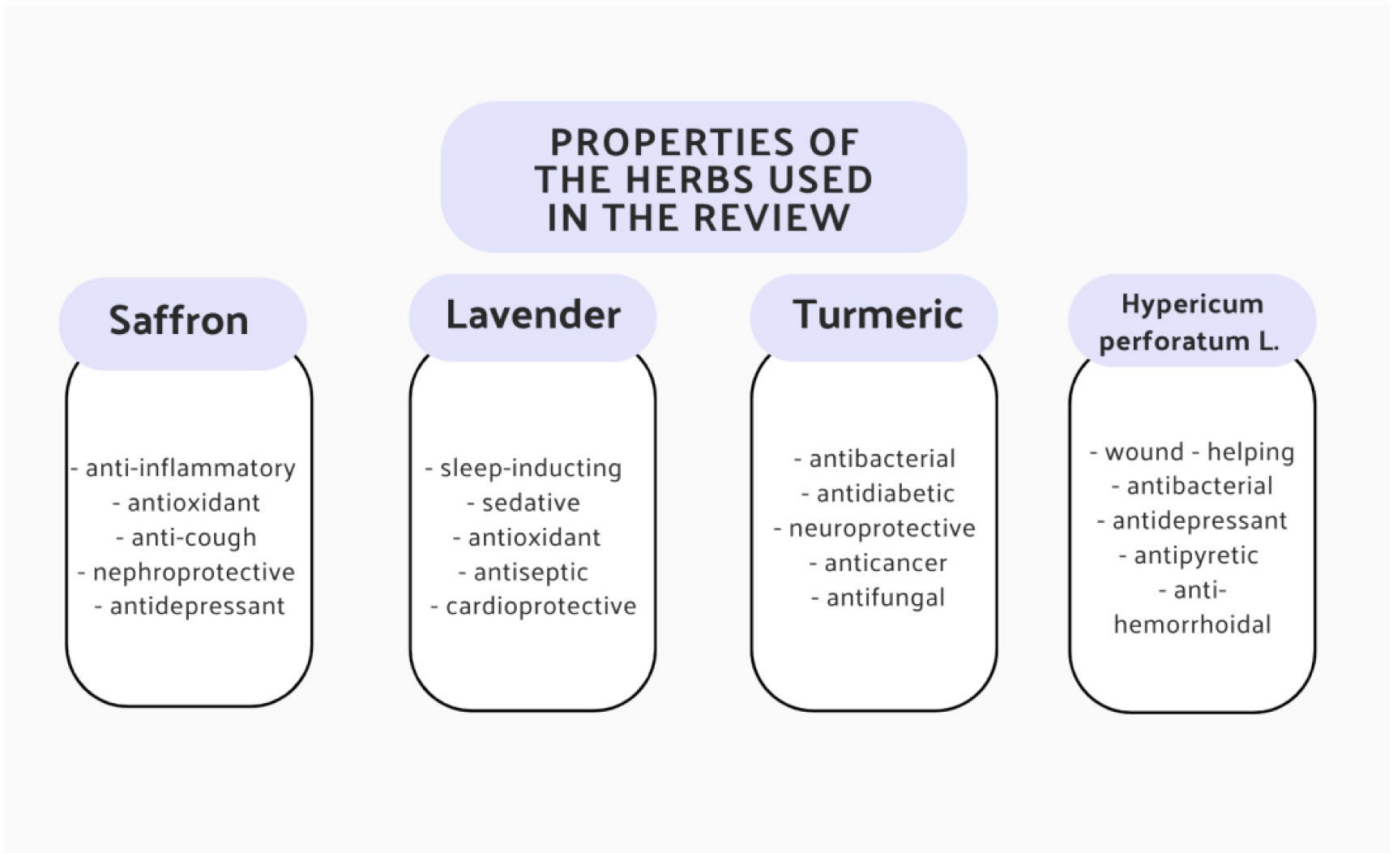
Summary of all the medicinal effects of saffron, lavender, turmeric and *H. perforatum L.* (20, 24, 26, 35).

**Figure 3** presents all properties of the herbs described above.

## 4 Phytochemicals in clinical researches

### 4.1 Saffron (*Crocus sativus L. stigma*)

*Crocus sativus L. stigma*, commonly known as saffron due to the presence of characteristic compounds, is a plant showing potential antidepressant effects as a result of regulation of the serotonergic and norepinephrine and dopaminergic systems, transmitters whose secretion is impaired in depression (36). *C. sativus* extracts are thought to inhibit the reuptake of serotonin, dopamine and norepinephrine, but the main antidepressant effects are attributed to crocin and crocetin. There are also studies showing that safranal has an affinity for 5HT1AR/5HT2AR receptors (37, 38). Specifically, crocin has been shown to act as a noncompetitive inhibitor of human MAO-A and MAO-B as a result of binding to the allosteric site of the enzyme (39). Molecular mechanisms signify that crocin exerts anti-inflammatory effects by reducing the expression of the mRNA of tumor necrosis factor α (TNF-α), interleukin 1β, interleukin 6 or interferon γ (IFN-γ) (39).

In studies on the patient, saffron has good results in a placebo-controlled studies. In the first reported randomized, double-blind, controlled study, 54 adults aged 18-70 years were included. They were divided into two main groups of 27 people each, the research group received 50 mg of dried saffron in capsules twice daily, the control group received a placebo (40). The treatment effect was measured after 12 weeks using the Beck Depression Inventory (BDI). The difference between the endpoint and the baseline in the test was -8.65 in the saffron group and -5.46 in the placebo group (40).

Another double-blind randomized trial was conducted in breastfeeding mothers suffering from postpartum depression (41). The study lasted two months and included mothers over 18 years of age who had given birth to a live baby, had no history of substance abuse, had not previously taken mood-altering medications, and had no previous history of mental disorders or depression. 60 breastfeeding mothers were randomly assigned to two groups: they received 30 mg of saffron daily (n = 30, 15 mg/twice daily) or an equivalent placebo dose (n = 30) for 8 weeks. The Beck Depression Inventory – II scale (BDI-II) (21 questions, multiple choice) was used to evaluate the results, which is one of the most frequently used psychometric tests to measure the severity of depression (effectiveness - 90%). The BDI-II is scored as follows: 0-13 points – no or minimal depression, 14-19 – mild depression, 20-28 – moderate depression, and 29-63 – severe depression. At last follow-up, 23 of 30 participants in the saffron group had depression scores indicating that they no longer suffered from clinical depression (BDI-II score ≤ 10). Which means that at the last evaluation, 77% of the saffron group was in remission. This value was significantly higher than in the 43% of the placebo group who were in remission (p < 0.001) (41). No adverse events were observed in the placebo group. In the saffron group, patients also did not complain of any discomfort or significant side effects, but they did report several inconveniences presented in **Table 1**.

**Table 1:** Side effects reported by patients after taking saffron (41).

Adverse event	Saffron patients (%)	Placebo patients (%)
Lack of sleep	3.3	0
Oversleeping	3.3	0
Low breast milk level	6.2	0
Gastrointestinal disorders	6.2	0

Another double-blind randomized study involved 121 people aged 18-77 years who reported low mood, but had not previously been diagnosed with depression or other comorbidities (42). Participants who self-reported depressed mood but were not diagnosed with depression were enrolled and randomized to receive saffron extract - affron^®^, 22 mg/day (n=42) or 28 mg/day (n = 41) or placebo (n = 38) for 4 weeks. The results were presented using the Profile Of Mood States (POMS) test (high vigor scores reflect good mood or emotions, and low scores in the remaining subscales reflect bad mood or emotions, when it comes to tension, depression, anger and fatigue, the higher the score, the worse the patient’s well-being): in the placebo group, result of tension, depression, anger, fatigue, and vigor were respectively: -1, -1.5, - 0.5, -2, -5, in turn in the group with 28 mg/day of saffron, respectively: -4, -9, -6, -5 and 4. Dose of saffron 22 mg/day: -4, -4, - 3, -3 and 2. The results clearly indicate an improvement in well-being in subjects taking saffron (42).

It is important to note that depressive disorders are also associated with elevated levels of TNF-α (43). Numerous studies have shown that healthy patients have lower levels of pro-inflammatory cytokines than depressed patients (43). The exact mechanism of crocin’s anti-inflammatory action is not known, but it is thought to act by inhibiting the nuclear factor kappaB trial (NF-*κ*B) (44).

In other test patients were allocated to two groups: the first group of 26 subjects received 15 mg of crocin and the second group of 27 subjects received a placebo, twice daily for 8 weeks. Both groups of subjects also took methadone in syrup form (45). 15 ml of blood was collected from each patient before and after the study and the expression of TNF-α genes in lymphocytes was compared by isolating RNA material from the samples. No adverse effects of crocin were observed in patients in the first group. In patients in the control placebo group, the amount of TNF-α was 1.1 units, which dropped to 0.8 units after crocin use. Patients themselves reported a slight improvement in well-being. In addition, the occurrence of interleukin 8 and 10 decreased, contributing to the effects of oxidative stress (45).

### 4.2 Lavender

Another herb is lavender. Although its exact mechanism of action is not known, it has been suggested that it may enhance the action of GABA in the amygdala (46). In addition, lavender oil has a modulating effect on the N-Methyl-D-aspartate receptor (NMDAR) (46). NMDAR is the receptor responsible for increasing synaptic plasticity activity (47). In the frontal cortex of suicide victims, NMDAR binding is significantly reduced, and expression of the receptor itself is severely reduced in depressed human subjects (47). Increased NMDAR activity in excitatory neurons activates serine/threonine protein kinase (Akt/mTOR) signaling, which regulates the initiation of protein translation and results in an increase in proteins required for synaptic plasticity in the frontal and prefrontal cortex, which may result in a reduction in depressive symptoms. In addition, mTOR may be specific for rapid-acting antidepressants, as drugs such as fluoxetine do not act through kinase signaling (48). What’s more, lavender helps renew neurons in the brain, which can be shown to increase bromodeoxyuridine (BrdU) levels. Bromodeoxyuridine is an exogenous pyridine analogue used as a marker for DNA synthesis. BrdU is incorporated during the cell cycle into the DNA of cells, including neurons (49). Sánchez-Vidaña et al. made an experiment, where rats that inhaled with 1 ml of 2.5% lavender oil solution had more BrdU-positive cells in the brains than control group rats (50). This shows the positive effect of lavender oil on nervous system cell proliferation (50).

They also tested effect of lavender oil on serum levels of brain-derived neurotrophic factor (BDNF). Compared to the control group, BDNF levels were much lower than in rats brains with lavender inhalation (50).

BDNF, is a factor that contributes to the normal development and regulation of plasticity of synapses, particularly glutamatergic and GABA-ergic synapses (51). Reduced BDNF exacerbates depression-like behaviors - selective BDNF deactivation in the hippocampus resulted in depression-like disorders (52). BDNF, by binding to the tropomycin receptor kinase B (TrkB), causes polymerization of this receptor, and its tyrosine residues inside the cell become phosphorylated (53). Thus, the activated receptor activates several enzymes and their dependent pathways: the first enzyme is phosphatidylinositol 3-kinase (PI3K) - it shows anti-apoptotic effects and modulates NMDA receptor-dependent synaptic plasticity (53). The next protein is phospholipase C gamma (PLC-γ) - increases synaptic plasticity by activating CAM kinases and protein kinase C, subsequently causing an increase in 1,2 - diacylglycerol and Ca 2+ calcium ions, and the final enzyme is mitogen-activated protein kinase (MAPK), which regulates protein synthesis during neuronal cell differentiation and is required for the activation of ERK1/2 and CERB, which are required for cytoskeleton synthesis and neuronal branching in the hippocampus (54).

To confirm the antidepressant effect of lavender on humans, an 8-week, randomized, double-blind clinical trial was conducted. 50 adult patients aged 18-65 took part in the study (55). They had to meet the criteria Diagnostic and Statistical Manual of Mental Disorder or have a score in Hamilton Rating Scale for Depression (HAM-D 17-item) between 8 and 24 (mild and moderate depression). These patients were divided into 3 groups, so that groups 1, 2 and 3 consisted of 17, 17 and 16 patients, respectively. Group 1 received *L. angustifolia* at a dose of 1 g/day, group 2 received fluoxetine, and group 3 received *M. officinalis* - 1 g/day. Patients were assessed on the basis of Hamilton Depression Scores at weeks 2, 4 and 8. The results in the first group were 17, 13, 10 points, respectively, in group 2: 16, 13, 8, and in group 3: 17, 14 and 11 points, which, however, was not statistically significant. The following side effects occurred in patients with lavender: headaches (2 people), increased appetite (3 people), nausea (1 person), dizziness (1 person) (55).

There have also been several human studies proving the effectiveness of lavender aromatherapy. The first study involves people over 60 years of age (56). Patients were divided into three groups of 20 people each - the first group received a massage with 5 ml of lavender oil (twice a week for 8 consecutive weeks), the second group received lavender essential oil inhalations of 50 µl mixed with 10 ml of water again twice a week for 8 weeks, and group 3 did not receive any form of aromatherapy. The Geriatric Depression Scale Short Form (GDS-SF) and the Patient Health Questionnaire-9 (PHQ-9) were used to measure outcomes, and 5-hydroxytryptamine (5-HT) levels were also assessed both before and after the test (56). Results in the essential oil groups performed at 8 weeks post-treatment, 6 weeks post-treatment and 10 weeks post-treatment showed a reduction in depressive moods, while in the control group, test results remained the same before and after the experiment. As for the serum levels of 5 - HT in the subjects, compared to the controls, they were significantly higher than before the study - in the massage group the initial level was 32.22 ng/mL, after therapy it was 40.52 ng/mL, in the oil inhalation group before the study the 5 - HT level was 29.96 ng/mL and after the study 36.49 ng/mL, in the control group respectively: 34.87 and 30.79 ng/dL (56). The results of measuring 5 - HT are shown in **Table 2**.

**Table 2:** Level 5 - HT in patients’ serum before and after lavender oil treatment (56).

Group	Level 5 – HT before the test [ng/ml]	Level 5 – HT after the test [ng/ml]
Control	34.87	30.79
Massage with lavender oil	32.22	40.52
Inhalation with lavender oil	29.96	36.49

The effect of lavender oil on postnatal depression was also studied. The study involved 140 women from an obstetrics and gynaecology department. They were randomly assigned to two groups - a control group using classical therapy and a study group using inhalations of 3 drops of lavender oil every 8 hours for the next 4 weeks (57). After 2 weeks, the first and third month, the patients were assessed on the 21-item depression, anxiety and stress scale and the Edinburgh stress, anxiety and depression scale. Patients’ mean anxiety scores were significantly lower in the study group than in the control group, and the frequency of depression distribution percentages after 2 weeks, 1 and 3 months in the control group were 25%, 21% and 17%, respectively, and in the study group 13%, 2% and 3%, respectively (57). Lavender reduced anxiety and depression in postpartum patients.

Another single-blind study was also conducted in 60 elderly people (30 people in each group) to test the effects of lavender tea on anxiety and depression (58). One of the conditions for inclusion was depression score between 14–28 (slight or moderate depression) and no use of antidepressants. The average age of the patients was 66 years. The intervention involved the use of 2 g of lavender tea bags, which were prescribed to be consumed twice as a decoction in the morning and evening. The duration of the intervention was 2 weeks. Patients were randomly assigned to groups. Scores were collected from the BDI – II and The State-Trait Anxiety Inventory (STAI). The results were as follows: the average depression scores in the control group were 18.40 ± 1.81 points before the study and 18.33 ± 1.84 points after the study. However, in the research group, the results were: before the test 17.80 ± 1.49 points, and after the test 16.33 ± 1.49 (P<0.001). In turn, in the anxiety score - in the control group before the test, the average score was 45.50 ± 6.95 points, and after the test 46.47 ± 6.77. In the study group: before the study - 45.47 ± 7.28 points, and after the study 40.07 ± 4.80 (P<0.001) (58). Based on the above study, it can be seen that lavender statistically significantly reduces depression and anxiety indicators in elderly patients.

### 4.3 Turmeric (*Curcuma longa*)

A herb worthy of consideration in the treatment of depression is also turmeric (*Curcuma longa*), or rather the polyphenol curcumin contained in it. It is a plant that has long been known and used in Indian folk medicine. Although the exact mechanism of action is not known, it appears that curcumin stimulates the release of serotonin and dopamine, inhibits MAO activity and regulates the hypothalamic-pituitary-adrenal (HPA) axis (59). In depressed patients, the HPA is up-regulated and negative feedback control is reduced. Corticotropin-releasing factor is hypersecreted from the hypothalamus and causes increased release of adrenocorticotropin (ACTH) from the pituitary gland, resulting in elevated cortisol levels in depressed patients (59).

According to studies, an estimated 40 to 60% of depressed patients have elevated blood cortisol levels (60). Curcumin lowers corticosterone levels by inhibiting the action of ACTH - in studies on bovine cells *in vitro*, in a control trial, administration of 2 nM ACTH increased cortisol levels up to 800 ng/106 cells, while curcumin was added to ACTH at doses of: 1, 5, 10 and 20 µM, which reduced cortisol concentrations to: 650, 100, 50 and 45 ng/106 cells and by inhibiting 11β-hydroxysteroid dehydrogenase involved in the conversion of cortisol to cortisone and vice versa (61).

In human studies, eighty patients aged 18 - 70 years, with a body mass index (BMI) of 25 - 30 kg/m2 took part in a randomised, double-blind study. 80 patients were equally divided into two groups (n = 40): one group received placebo and the other received curcumin 800 mg after lunch for 56 days. The placebo group had an average cortisol level of approximately 23 mcg/dL after 56 days, and the curcumin group had an average cortisol level of approximately 18 mcg/dL (62).

Another research also show that curcumin reduce the level of cortisol. A double-blind, randomized study was conducted on 100 adult men aged 31–59 years (63). Participants were divided into two groups: one group received 2 capsules containing 1000 mg of curcumin, and the other group received a placebo in the form of soy powder, once daily for 6 weeks. During the first week of the study, patients were also given escitalopram at a dose of 5–15 mg. After completing the capsules, the participants’ salivary cortisol levels were measured: in the placebo group, the level was 83.04 ng/ml, and in the research group 54.30 ng/ml. Curcumin significantly reduced the cortisol levels in the subjects. In addition, the level of BDNF in the serum of patients was also examined: in the control group, the BDNF level was 310.38 pg/ml, and in the group with curcumin - 407.28 pg/ml, which proves that curcumin acts on multiple levels, contributing to the restoration of homeostasis in the patients’ organism (63).

In addition, curcumin quiets inflammation in the body. In patients with depression, meta-analyses found statistically significant elevations of Il - 6 in cerebrospinal fluid (CSF) particularly in suicide attempters - in the control group, Il - 6 levels were around 11 pg/mg protein, and in adolescent suicide victims about 25 pg/mg protein, while a study of CRP levels in CSF from a total of 13 541 patients found that 58% had elevated CRP >1 mg/l and 27% had CRP > 3 mg/l (64, 65). Besides, in a meta-analysis presented by Osimo et al. TNF-α in the serum of depressed patients reaches higher levels than in healthy subjects (66). Attention should also be paid to the kynurenine pathway (KYN), which is activated by inflammatory cytokines such as the just-mentioned Il - 6 and TNF. Thanks to KYN, tryptophan (TRP) is broken down into KYN metabolites, leading to the formation of nicotinamide adenine dinucleotide (NAD+) - KYN is responsible for 95% of tryptophan metabolism. Importantly, tryptophan is a precursor amino acid for the formation of serotonin (67). Through increased levels of inflammatory markers, the KYN pathway is over-activated and this leads to decreased serotonin levels and depressive disorders.

To proof the anti-inflammatory effect of curcumin 67 patients were randomly assigned to two groups; the study group received 2 curcumin capsules twice daily - a total of 150 mg of curcumin - and the control group received placebo for 8 weeks. The subjects getting the placebo had a serum Il - 6 concentration of 3.57 pg/mL ± 2.21, and the curcumin-treated patients had a serum Il - 6 concentration of 1.83 pg/mL ± 0.67 (49). Importantly, IL-1β and TNF-α are other inflammatory cytokines whose levels are reduced during curcumin administration. In the study by Yu Jing-Jie et al. already cited above, the levels of these cytokines in plasma were also examined after the patients in the research group were given 1000 mg of curcumin (2 capsules daily). In the placebo group (n = 50), the levels of IL-1β and TNF-α were 8.37 pg/mL and 70.04 pg/mL, respectively, in the curcumin group (n = 50), 6.69 pg/mL and 52.65 pg/mL, respectively (63).

A recent study examined the effectiveness of turmeric in severe depression. A randomized, double-blind study was conducted in which 123 people aged 18-65 with major depressive disorder took part (68). The subjects were divided into 4 groups, they took the following substances: placebo, curcumin extract in small doses (250 mg twice a day), curcumin extract in large doses (500 mg twice a day) or a combination of curcumin extract in small doses with saffron (15 mg twice daily) for 12 weeks. The measure of treatment effectiveness was determined on the basis of the Depressive Symptomatology self-rated version (IDS – SR 30). Subjects were asked to complete forms at baseline, weeks 4, 8, and 12. In the placebo group, the results were 52.25, 46.52, 48.06 and 49.99 points, respectively. In the turmeric 500 mg/day group: 53.54, 48.93, 46.85 and 42.73 (P<0.001). In the group with turmeric at a dose of 1000 mg/day: 54.73, 49.30, 46.03 and 44.07 (P<0.001). In the group of subjects with turmeric and saffron, the results were similar to those in the group with turmeric at a dose of 1000 mg/day. The respondents also reported several side effects: diarrhea (7 people), spicy taste (9 people), headaches (5 people) (68). Based on the results of this study, it can be seen that turmeric has beneficial antidepressant effects.

**Figure 4:**
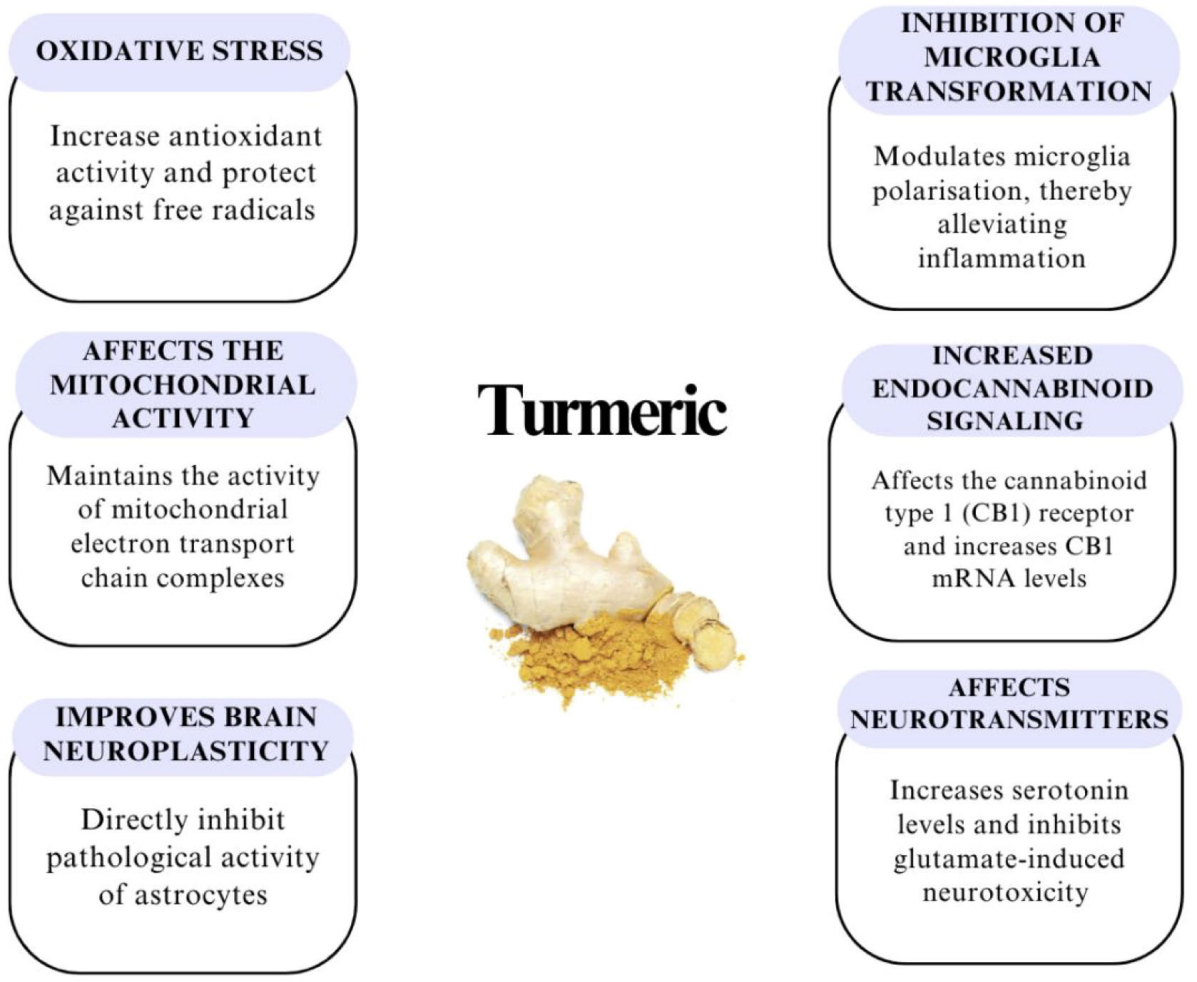
Other potential effects of curcumin in combating depression – acts as an antioxidant, has a positive effect on neuroplasticity in the brain, protects mitochondria and therefore reduces oxidative stress, regulates the endocannabinoid system – influences the modulation of inflammatory reactions and anxiety, and regulates serotonin, dopamine and glutamate (49, 61, 69).

**Figure 4** shows possible mechanisms of action of turmeric.

### 4.4 *Hypericum perforatum L.* (St. John’s Wort)

This is another interesting plant in the treatment of depression. The antidepressant effect is achieved by several mechanisms: hypericin inhibits the activity of MAO and flavonoids inhibit MAO through structurally similar to its inhibitors.

The mechanism of action of hypericin is based on the influence on cytochrome (CYP) enzymes, maintaining the appropriate amount of serotonin and silencing neuroinflammatory signaling pathways (70). Importantly, it has been shown that the modification of mRNA N6 – methyladenosine (m6A) disrupts the neurobiological mechanism in the brains of patients with depression and that the effectiveness of tricyclic drugs (TCA) is associated with modifications of m6A. m6A is important because its stable modifications contribute to the proper development of the brain and, conversely, disturbances in this process can lead to the development of disorders, including depression (71).

Hyperforin, in turn, inhibits the reuptake of serotonin, noradrenaline, dopamine and GABA, and also inhibits catechol-o-methyltransferase (72). Inhibition of serotonin uptake in *in vivo* studies shows that it may be related to an increase in intracellular sodium concentration, as hyperforin interacts with Na+ channels or Na+/H+ exchangers. Another mechanism explaining the antidepressant effect is based on the modulation of intracellular calcium levels in mice by hyperforin, thereby potentiating the effect of lanicemine, which is an NMDA receptor antagonist. Importantly, hyperforin activates Ca2+ dependent signaling pathways involved in neuroplasticity. Other studies report inhibition of L-glutamate and GABA uptake (72).

To confirm the efficacy of St. John’s wort in treating depression, a double-blind, double-dummy, randomized phase III study was conducted. The study included 64 patients of both sexes, aged 18–70 years (73). The first group (n = 31) received 300 mg of St. John’s wort extract 3 times a day, and the second (n = 33) 20 mg of paroxetine for 6 weeks. The efficacy of the therapy was assessed using the Hamilton Depression Rating Scale (HAM – D), where 8–16 points are assessed as mild depression, 17–23 as moderate, and 24 and above as severe. Patients included in the study had to have a score of ≥22 points. Patients were assessed on days 7, 14, 28, and 42 of therapy. The change in the total score in the above-described scale in the St. John’s wort group on day 7 was -6.1, on day 14 -10.2, on day 28 -14.2, and on day 42 -16.4. In the paroxetine group, respectively: -3.0, -7.5, -10.1, -10.1 (all results with a p value of <0.05). In light of the presented results, it can be seen that St. John’s wort therapy seems to be more effective than a classic antidepressant, with complete remission in the paroxetine group being 14 subjects, and in the St. John’s wort group 22 subjects (p<0.05). 19% of patients treated with St. John’s wort reported adverse effects such as abdominal pain or constipation or dizziness, and in the paroxetine group 61% reported constipation, headaches, drowsiness, hand tremors, loss of libido, anxiety or sleep disorders (73). To summarize the above study, St. John’s wort was found to be more effective in the treatment of moderate to severe depression and caused fewer side effects compared to paroxetine.

In another double-blind randomized study, 51 people aged 18–55 years with mild to moderate depression and no other psychiatric problems were randomly divided into two groups: one group received two capsules of St. John’s wort at a dose of 250 mg (n = 23) and the other group received two tablets of fluoxetine 20 mg (n = 28) for 8 weeks (74). The HAM – D scale was used to assess the results. The result at the beginning of the study in the fluoxetine group was 27.64, and with St. John’s wort 27.22. After 4 weeks in the fluoxetine group the result was 20.64, and after 8 weeks 16.93. In the St. John’s wort group the HAM – D results after 4 weeks were 21.13, and after 8 weeks 17.0 (p<0.001). 50% of patients in the fluoxetine group reported side effects, and in the St. John’s wort group 30.4% of them, these were mainly nausea, dry mouth or dizziness. The above study does not show significant differences in the effectiveness of St. John’s wort and fluoxetine, which means that both preparations are equally effective in the treatment of depression (74). Regarding adverse events associated with the use of St John’s wort in general, a European study of 3250 patients found that only 2.4% of patients reported adverse events (75). **Table 3** presents the percentage of the most common side effects.

**Table 3:** Statement of side effects when using St. John’s wort (75).

Side effect	Percentage share [%]
Gastrointestinal irritations	0.6%
Allergic reactions	0.5%
Fatigue	0.4%
Restlessness	0.3%

This proves that the use of natural medicines is burdened with less persistent side effects than traditional medicines, and the percentage of their occurrence is much lower than in the case of classical pharmacotherapy, without losing its effectiveness. **Table 4** were prepared to summarize the studies collected in the review.

**Table 4:** A summary of the works presented in the review.

Author and year	Population	Intervention	Study design	Results and quality assessment
Mazidi M. et al. (40), 2016	54 patients	50 mg of saffron capsule twice a day	RCT	Reducing score in BDI, high quality (P<0.05)
Tabeshpour J. et al. (41), 2017	60 women	30 mg/day of saffron	RCT	Reducing symptoms of depression, high quality (P<0.001)
Kell G. et al. (42), 2017	128 patients	affron^®^ at 28 mg/day	RCT	Reducing sadness and improving vigor, high quality (P<0.001)
Ghaderi A. et al. (45), 2019	53 patients	15 mg of crocin twice a day	RCT	Decrease in TNF α levels in peripheral blood serum, high quality (P<0.05)
Araj-Khodaei M. et al. (55), 2020	50 patients	*L. angustifolia* 2g/day	RCT	Decrease in anxiety and sadness levels, intermediate quality (P=0.192)
Xiong M. et al. (56), 2017	20 patients	Lavender essential oil inhalations of 50 µl	RCT	Increase in the level of 5-HT in blood serum, hight quality (P<0.001)
Kianpour M. et al. (57), 2016	140 women	Three drops of lavender oil 3 times a day	RCT	Decrease in anxiety and depression rates, high quality (P<0.05)
Bazrafshan MR. et al. (58), 2020	60 patients	2 g lavender tea bags a day	RCT	Decrease in anxiety and depression levels, high quality (P<0.001)
Cicero AFG. et al. (62), 2020	80 patients	Curcumin 800 mg after lunch	RCT	Decrease level of cortisol in serum, high quality (P<0.05)
Tabrizi R. et al. (49), 2018	67 patients	2 curcumin capsules twice daily - a total of 150 mg of curcumin	Metaanalysis	Decrease in the level of Il - 6 in blood serum, hight quality (P<0.001)
Yu JJ. et al. (63), 2015	100 patients	2 capsules containing 1000 mg of curcumin	RCT	Decrease in salivary cortisol levels, high quality (P<0.001)
Lopresti AL. et al. (68), 2017	123 patients	500 mg of curcumin twice a day	RCT	Significantly greater improvements in depressive symptoms, high quality (P<0.012)
Seifritz E. et al. (73), 2016	64 patients	300 mg of St. John's wort extract 3 times a day	RCT	Reduced score in Hamilton Depression Rating Scale, high quality (P<0.05)
Sadeghi A. et al. (74), 2023	51 patients	Two capsules of St. John's wort at a dose of 250 mg	RCT	Reduced score in Hamilton Depression Rating Scale, high quality (P<0.001)

## 5 Discussion

Many of the medicinal plants presented in this review undoubtedly affect the activity of the central nervous system in an antidepressant way, which is most important in our work, and anxiolytic. The selection of the plants we mentioned was not accidental. In order to provide the latest data, we tried to select works from a relatively narrow time range, but in the period 2015-2024 there are not many studies that would determine the specific action of the active substances found in these plants. Most of them are only assumptions based on studies on rodents. In addition, there are few studies currently conducted on patients, perhaps this is not as popular a topic as it should be. This issue certainly requires further work and development, because in a plant such as lavender, a specific phytochemical that could improve the well-being of patients has not yet been identified. Additionally, the maximum dose of taking the mentioned herbs has not been specified anywhere. Only curcumin is known to have a maximum dose of 8 g/day, standard 200 mg to 6 mg per day for up to 8 months, while for lavender it is only known that the effective dose is 80-160 mg (76). Lavender oil can be used by giving a single or a few drops, making 20 to 120 mg, diluted, while in tea form, 1 or 2 teaspoons, but in the study cited, Xiong et al. gave only 5 mg twice a day, a lower dose, with a noticeably positive effect on patients’ well-being. With saffron, the best therapeutic effects are shown by 20-400 mg per day, with a maximum daily dose of 5 g and lethal dose equals 20 g. There is no information about St. John’s wort. Besides, the treatment regimens are not strictly defined, the authors of the cited studies also use herbal therapies at different time intervals, they are sometimes 4 sometimes 8 weeks. All herbal preparations should be taken orally, except for lavender, which can also be used as aromatherapy or massage oil.

Importantly, there are no studies on the efficacy AND safety of the mentioned plants during pregnancy, but the use of St. John’s wort during lactation has been described. A 33-year-old patient with postpartum depression who was breastfeeding was given St. John’s wort herb for use (77). When the active substances of St. John’s wort were determined in breast milk, it was found that hypericin was below the detection threshold, while hyperforin was in only small amounts - from the determination of the below normal to a maximum of 2.76 ng/ml. After determining these active substances in the child’s blood, it turned out that both hypericin and hyperforin were below the detection threshold. No side effects were observed in the mother as in the child. However, this is only one study of this type, so the issue of the use of herbs in depression should be developed. It is important to remember the abortive effect of saffron, so its use is not recommended during pregnancy. There was also no mention of how phytotherapy works on underage and younger patients. Given that these are natural therapeutic agents perhaps they would be more readily used in children and adolescents than strong antidepressants. However, this requires that additional clinical studies be undertaken.

Side effects are also important in the use of natural remedies. While taking saffron, only 3.3% of patients had sleep problems in the form of insomnia or hypersomnia, and 6% had gastrointestinal problems (41). The use of lavender in only 7 people out of 50 in experienced minor side effects such as mild headaches or dizziness, and decreased appetite - as a rule, when using antidepressants, patients report increased appetite, which contributes to weight gain (55). St. John’s wort herb in only 30.4%, compared to 50% of patients taking fluoxetine, complained of side effects of the herb such as dry mouth or dizziness (75). When taking classic SSRIs in a study of 100 patients, as many as 64% had bloating, 59% complained of lethargy, 45% weight gain, and 39% had hyperhidrosis not previously present (78). Side effects of plants are much less frequent and milder than classical pharmacotherapy, so perhaps in the future it will be increasingly common practice to administer a lower dose of SSRI in combination with lavender or saffron?

If we talk about drug combinations, unless there are contraindications in combining SSRI drugs with herbs, which researchers do in many of the studies mentioned above, there aren’t many articles on their interactions with other drugs. Synergistic effects between medicinal plants such as turmeric (*Curcuma longa*), saffron (*Crocus sativus*), lavender and drugs used to treat depression are increasingly being studied due to the growing interest in combination therapies. Based on the review presented, research suggests that curcumin may enhance the effectiveness of antidepressant drugs such as fluoxetine by modulating neurotransmitter pathways and reducing inflammation in the brain. Lavender, on the other hand, may support the effects of antidepressants by helping to reduce anxiety symptoms that often accompany depression. Hyperforin is believed to be a potent inducer of cytochrome 450, so it may modulate the effectiveness of drugs that affect this cytochrome (75). It is said that in order to avoid unwanted side effects due to interactions between St. John’s wort and other drugs, it is recommended to take a maximal 1 mg of hyperforin per day (79). Is this a sufficient dose? Given that in the studies, 250 mg of St. John’s wort was the lowest dose that patients look and in them the hyperforin content is estimated to be roughly 7.5 mg, avoiding interactions may be problematic. On the other hand, it is not known how often they occur.

The positions of the Food Drug Administration (FDA) or the European Medicines Agency (EMA) on extracts from the mentioned herbs vary. Turmeric and lavender have been “recognized as safe” by the FDA (80, 81). As for St. John’s wort, the FDA has not approved it as a drug, further in the United States of America it is considered a dietary supplement, while saffron like lavender or turmeric is considered safe, when used as a spice or food coloring additive (82). Lavender has been approved by the EMA as an herbal medicine to relieve stress and anxiety (83). In the case of turmeric, the EMA has taken the position that turmeric can be used in herbal medicinal products for mild digestive problems, while saffron has not yet been evaluated by the Committee for Herbal Medicinal Products (HMPC) of the EMA (84). The fact that some herbs are not mentioned or studied by the FDA or EMA does not mean they are not effective in treating depression. Many of them, such as turmeric have been used in Chinese medicine and Ayurveda for many years. What more, NICE mentioned about St. John’s Wort in treatment of less severe depression, but not advice to use it, because of uncertainty about doses (9).

The plants mentioned in the review show a variety of mechanisms of action contributing to the improvement of the well-being of people who use them. The most interesting and multi-dimensional is turmeric. It inhibits inflammation, regulates the HPA axis, prevents oxidative stress and promotes the preservation of brain neuroplasticity, theoretically it is a counter to all the theories of the pathogenesis of depression that we have learned so far, but it is not known how it is in practice, but the research results seem to be quite promising. Lavender, in turn, affects the increase of BDNF, thanks to which the plasticity of synapses develops, which is disturbed in depression. Saffron, like St. John’s wort, inhibits the reuptake of serotonin and noradrenaline, while saffron additionally reduces inflammation. Nevertheless, there is little information about St. John’s wort and its specific mechanism of action, which provides a good opportunity to develop the subject in the future.

The studies presented by the authors show good efficacy of phytotherapy in depression. Most of the results are statistically significant, however, there are some, such as Araj-Khodaei M. et al., whose study results, despite demonstrating the effectiveness of using 2 grams of lavender per day, were not statistically significant (P=0.192). However, taking into account the entire review, it seems that it is possible to consider the introduction of herbs in the treatment of a mental illness such as depression, especially lavender inhalation. Such a procedure is not expensive or difficult for the patient to perform, and most importantly, it is a method of low invasiveness.

Moreover, it is important to remember that there are many types of depression, and each is different. The manuscript cited disorders from mild to severe according to the report of the authors of the works cited. At present, there is not enough research on phytotherapy for different degrees of depression or the use of herbs for each type of depression. This requires further research with well-diagnosed and classified patients.

Of course, nowadays, first-line medications should be used first, as there have been many more studies on their effectiveness. However, all of the plants presented offer a natural addition for treating depression. Perhaps they would work well in monotherapy for those who are set on using only natural treatments, or as an adjunct therapy to existing treatments to help reduce the dose of medications used. Adding the herbs mentioned in the review to the treatment of depression is in line with customizing individual therapy and allowing to sprout the requirements of patients. Considering recommendations of the European Society of Clinical Pharmacy for Mental Health, clinical pharmacy as a science and service can be useful for physicians and patients with mental illness (85, 86). It has clinical and economic benefits and may address some of the practice gaps (more effective use of drugs, pharmacy-education and others). The results of the review indicate the need for further research on the combination of both therapies (traditional pharmacotherapy and phytotherapy) to assess the effectiveness and safety of treating patients with depression while reducing the costs of treatment. European-wide need for standardised and effective interventions to optimise the use of psychotropic drugs in many patient groups (due to age, different categories of severity of depression symptoms, and the presence of various chronic health problems) and under different conditions. Many of the herbs already mentioned, such as saffron, lavender, and turmeric, also have the ability to help patients with anxiety, can be used in different forms, it affects different receptors, it’s making them an interesting option for a holistic approach to the patient’s mental health.

In light of the facts presented, the attending physician may consider using natural methods in therapy. Will the use of phytotherapy become more popular due to the research being conducted? Perhaps. It is certainly difficult to convince physicians to use natural methods, even though today patients insist on using natural remedies whenever possible. Moreover, it is not known how much of a given plant should be cut to obtain appropriate doses for patients that give the desired effect - it is worth mentioning that a larger amount of natural active substance is needed to obtain the same effect as in traditional treatment. Perhaps adding some of these plants, such as non-invasive inhalations or massages with lavender essential oil, to traditional antidepressants would improve the well-being of patients and possibly reduce side effects occurring when using regular pharmacology, which would improve the quality of life of patients. In summary, physicians need strong scientific evidence to make appropriate clinical decisions.

## 6 Limitations

In the above review, there are several factors that may limit the possibility of drawing hasty conclusions and using generalizations. One such limitation is the size of the study sample, these are not really large groups. The review was based on RCTs. Within psychiatry, this can represent a significant issue in assessing the effectiveness of treatments in the typically diverse patient populations presenting in practice (e.g. coexistence of other chronic health problems). Most patients, with mental illnesses, are not represented in typical RCTs, limiting evidence extrapolation. It is also not stated whether the patients take other medications, in some studies the condition for inclusion was not to have previously used antipsychotics or SSRIs, but what about the rest? The authors do not mention it.

Additionally, the time of using the preparations is relatively short, it is 6-8 weeks, sometimes 12. We do not know how natural preparations affect the body during longer use. Due to these limitations, we try to be cautious in interpreting the articles, and the dry facts and research results are presented.

An additional limitation were publications that we searched for only in English, however, when collecting materials we did not come across search results in other languages. We narrowed the search results to 2015 to provide the most up-to-date evidence on the use of herbal therapy in depression.

## 7 Conclusions

Plants such as turmeric, lavender, saffron and St. John’s wort, reveal effective mechanisms of antidepressant and anxiolytic effects, but many studies are conducted on the tests in rodents. Although phytotherapy itself is a rapidly developing field, its use in the treatment of depression still needs to be confirmed in human studies. However, the experiments conducted so far seem to be a good start to enter a new era of medicine, in which classical treatment will be supported by phytotherapy. Due to the small number of clinical trials, more studies should be carried out, taking into account the number of participants, the severity of depression symptoms, the coexistence of others chronic health problems, and thus deepen the knowledge of the mechanisms of action, analyse the efficacy and safety of the plants mentioned. Integrating natural treatments with classical pharmacological therapies may be a promising avenue for improving the quality of life of patients with mood disorders and reduce treatment costs.

## Data Availability

The original contributions presented in the study are included in the article/supplementary material. Further inquiries can be directed to the corresponding author.
